# Observation of the Epithelial Cell Behavior in the Nasal Septum During Primary Palate Closure in Mice

**DOI:** 10.3389/fphys.2020.538835

**Published:** 2020-10-02

**Authors:** Sayuri Yamamoto, Hiroshi Kurosaka, Jiro Miura, Gozo Aoyama, Safiye Esra Sarper, Ayaka Oka, Toshihiro Inubushi, Kohei Nakatsugawa, Yu Usami, Satoru Toyosawa, Takashi Yamashiro

**Affiliations:** ^1^Department of Orthodontics and Dentofacial Orthopedics, Graduate School of Dentistry, Osaka University, Osaka, Japan; ^2^Division for Interdisciplinary Dentistry, Dental Hospital, Osaka University, Osaka, Japan; ^3^Department of Biological Sciences, Graduate School of Science, Osaka University, Osaka, Japan; ^4^Department of Oral Pathology, Graduate School of Dentistry, Osaka University, Osaka, Japan

**Keywords:** nasal septum, cleft palate, live imaging, craniofacial abnormalities, epithelium, organ culture

## Abstract

Epithelial fusion is critical in palatogenesis, and incomplete fusion results in various type of facial cleft, depending on the region that fails to fuse. In mammalian palatogenesis, the bilateral secondary palatal processes fuse in the middle of the face to form the secondary palate. Later, the dorsal side of the secondary palatal shelves fuses with the nasal septum to complete palatogenesis. Importantly, the anterior border of the secondary palatal shelf fuses with the primary palate, which is located at the anterior and ventral border of the nasal septum. While numerous studies have investigated the mechanism of fusion between secondary palatal shelves, very little is known about how the primary palate touches and fuses with the secondary palatal shelves. In this study, we investigate the possible epithelial cell behaviors on the surface of the primary palate using palatal explant cultures of *K14-GFP* mice. A time-lapse observation of the GFP-labeled epithelium and an SEM analysis revealed that the extrusion epithelium appeared at the region corresponding to the fusing area and expanded rostrally on the nasal septum surface in the absence of the secondary palatal processes. Unlike on the secondary palate surface, cellular migration and subsequent autonomous mesenchymal exposure were not evident on the nasal septum or the primary palate. TUNEL staining revealed that these extrusion epithelia were undergoing apoptosis. These findings indicated that extrusion with apoptosis was autonomously initiated at the presumptive region of the fusion without contact with the opposing secondary palate.

## Introduction

Palatal fusion is essential for obtaining tissue continuity of facial processes from different embryonic origins. The palate is derived from both the primary palate and the secondary palate. The secondary palate fuses with the nasal septum, which is continuous with the primary palate (Ferguson, 1988). The primary palate and nasal septum are derived from the ventral protrusion of the frontonasal process, whereas the secondary palate develops by the outgrowth of the bilateral maxillary process (Hilliard et al., 2005). Palatal fusion occurs at the midline of the secondary palate following bilateral outgrowth of the maxillary process. Thereafter, in humans, the anterior and dorsal parts of the secondary palate fuse to the primary palate, and the posterior and dorsal portions of the secondary palate fuse with the nasal septum. On the other hand, in mice, fusion of the nasal septum to the palate only occurs in the most anterior region, and the posterior region would remain a common nasal passage (Ferguson, 1978; Yu et al., 2017). After these processes contact, the intervening epithelium between the growing shelves merges to form an epithelial seam, which must be removed to complete the fusion (Ferguson, 1988; Bush and Jiang, 2012). Most previous studies focused on palatal fusion between bilateral secondary palates; fewer studies have focused on the fusion between the secondary palate and the primary palate/nasal septum.

Multiple studies have demonstrated that the migrating epithelium plays major roles in removing the epithelial seam, while other cellular mechanisms, such as apoptosis and epithelial mesenchymal transformation, are unlikely to play a role—in a strict sense—in this process (Carette and Ferguson, 1992; Griffith and Hay, 1992; Shuler et al., 1992; Cuervo and Covarrubias, 2004; Jin and Ding, 2006; Kim et al., 2015). Among these mechanisms, cell migration was first showed by confocal imaging of Dil-labeled cells (Carette and Ferguson, 1992) and by *in vitro* chimeric culture experiments using *K14-Cre; R26R* mice (Jin and Ding, 2006). A recent time-lapse imaging study succeeded in directly capturing the dynamic migration of the fluorescently labeled medial edge epithelium of the secondary palate (Kim et al., 2015). On the other hand, in unpaired palatal shelf cultures (Takigawa and Shiota, 2004), where one side of the shelf is removed, the medial edge epithelium (MEE) disappears without contact to the opposing palatal shelf and consequent mesenchymal exposure could be observed. Interestingly, our recent time lapse observation revealed such autonomous epithelial disappearance in unpaired palatal shelf cultures, in which it was triggered by dynamic migration of the MEE (Aoyama et al., 2019). Furthermore, the region of mesenchymal exposure to epithelial migration never expanded anteriorly beyond between the first and second rugae in unpaired palatal shelf organ cultures (Charoenchaikorn et al., 2009), indicating that the fusion mechanism differs between the anterior and posterior palate at this boundary. Taken together, our time-lapse direct observation of the secondary palate surface clearly identified autonomous and intrinsic programmed events, even in the absence of the contact of the opposing process *in vitro*.

Along the anterior-posterior axis, palatogenesis is differentially regulated at the molecular level (Hilliard et al., 2005; Li and Ding, 2007). Several transcription factors and/or signaling molecules (e.g., *Msx1*, *Bmp4*, *Shh*, *Fgf10*, *Fgf7*, and *Shox2*) are specifically expressed in the anterior regions and some mutant mice also showed anterior cleft palate. Although a different gene mutation caused anterior-specific cleft palate, it was of interest that the posterior border of the anterior cleft is localized at the 1^st^ rugae (Dudas et al., 2006; Xu et al., 2006; Lane et al., 2015; Sarper et al., 2018b, 2019). However, most of previous investigations have focused on palatal fusion of bilateral secondary palate, and mechanisms of fusion in the anterior region between the primary and secondary palate is still largely elusive.

We evaluated the cellular behavior of the epithelium on the primary palate and the nasal septum using *K14-GFP* mouse explant culture as a model (Vaezi et al., 2002). Time-lapse imaging and an SEM analysis demonstrated that epithelial extrusion appears specifically on the regions of palatal fusion and that these extruding cells expanded rostrally along the fusing regions. We also showed that these cells are undergoing apoptosis. Our findings have shown that this experimental model can be a unique tool for exploring the possible regulatory mechanism of apoptosis that occurs in palatal fusion between the primary and secondary palate.

## Results

### SEM Observation of Cultured Mouse Nasal Septum

**FIGURE 1:**
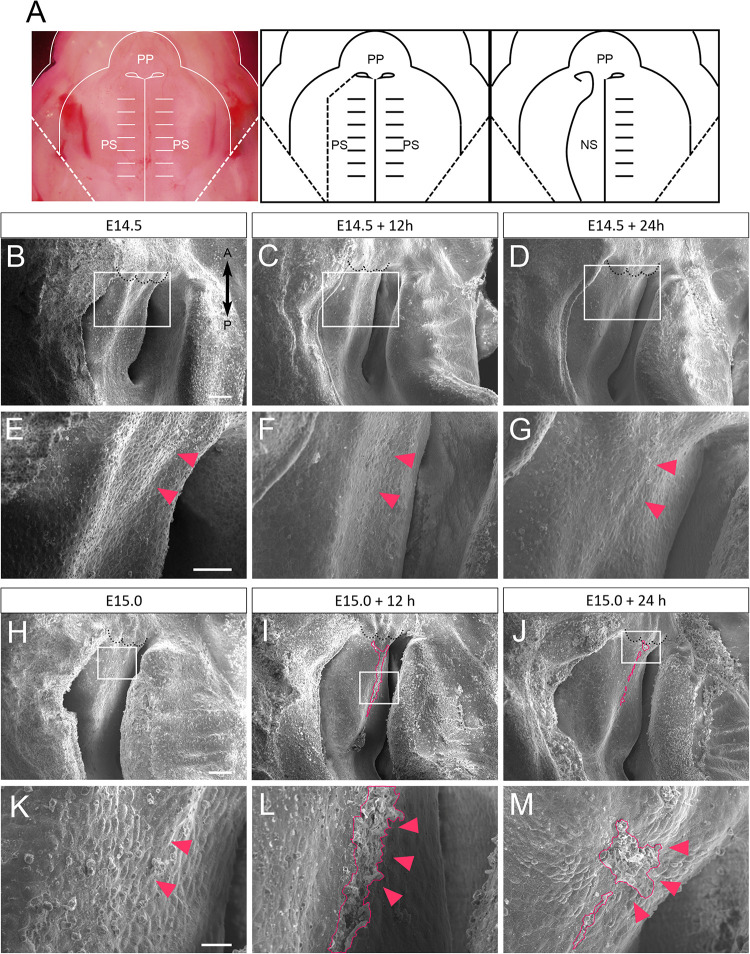
SEM observation of mouse nasal septum explants. **(A)** A schematic of the removal of the explants from the right palatal shelf from each maxilla. **(B–G)** Occlusal views of the WT mouse nasal septum at E14.5 **(B,E)**, after 12 h of culture **(C,F)**, and after 24 h of culture **(D,G)**. **(E–G)** Higher magnification views in the region indicated by white boxes in **B–D**. No protrusion of epithelial cells of the nasal septum occurred (red arrowheads). **(H–M)** Occlusal views of the WT mouse nasal septum at E15.0 **(H,K)**, after 12 h of culture **(I,L)**, and after 24 h of culture **(J,M)**. **(K–M)** Higher magnification views in the region indicated by white boxes in **H–J**. Protrusion of epithelial cells of the nasal septum occurred (red area, red arrowheads). Black dotted lines in **B–D** and **H–J** indicate the posterior border of the primary palate. A, anterior; P, posterior; PP, primary palate; PS, secondary palate; NS, nasal septum. Scale bars: **B** 100 μm (**B–D** same magnification); **E** 50 μm (**E–G** same magnification); **H** 100 μm (**H–J** same magnification); **K** 20 μm (**K–M** same magnification).

The secondary palatal process starts to fuse at the midline on E14.5, and then the anterior-most region of the secondary palate further fuses with the primary palate. At a similar developmental stage, the secondary palate also anterodorsally fuses with the nasal septum (Bush and Jiang, 2012). In order to investigate the detailed surface morphology of the anterior nasal septum during fusion with the secondary palate, we used a scanning electron microscope (SEM) to assess cultured maxillary complex explants. A dashed line in the Figure 1A schematic shows the plane of dissection for exposing the nasal septum during maxillary explant culture. Different embryonic time points were selected to observe stage-specific events of nasal septum development. At E14.5, the surface of the nasal septum showed a somewhat smooth surface (Figures 1B,E). Even after 12 h (Figures 1C,F) and 24 h (Figures 1D,G) of culture, the surface morphology of the nasal septum did not show noticeable differences. However, when we started culturing the explant from E15.0 (Figures 1H,K), extruding epithelial cells could be observed after 12 h of explant culture at the presumptive fusion site of the nasal septum and the secondary palate (Figures 1I,L) and also remained after 24 h of culture (Figures 1J,M). These results indicate that the nasal septum epithelium exhibits protrusive extruding behavior during the process of fusion with the secondary palatal shelves.

The explants were approximately 1500 μm in width, 1000 μm in length, and 700 μm in thickness (Supplementary Figure 3). Previous reports using a similar size of maxillary complex for organ culture showed certain cellular events that recapitulate the *in vivo* situation (Shiota et al., 1990; Takigawa and Shiota, 2004).

### Induction of Extruding Cells on the Epithelial Surface of Primary Palate Explants of K14-GFP Mice

**FIGURE 2:**
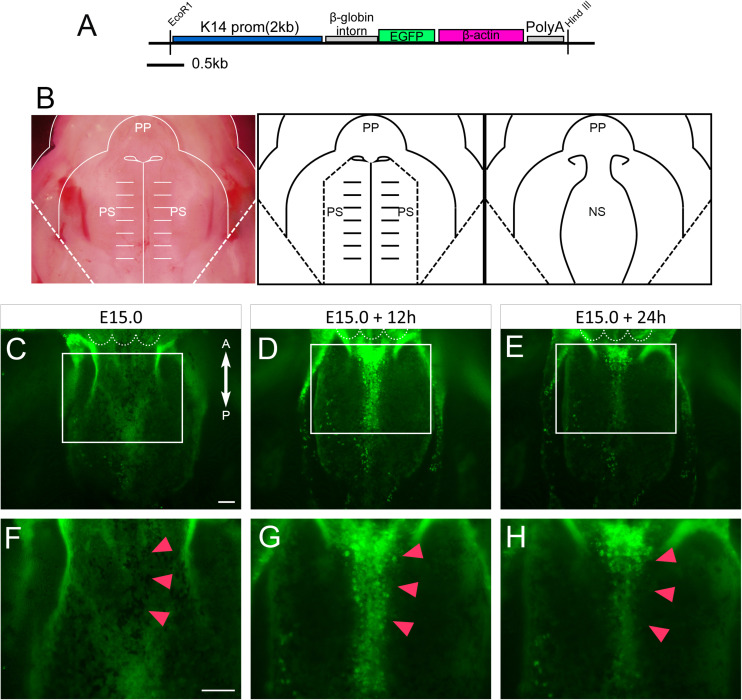
An anatomical analysis of the nasal septum of the *K14-GFP* mouse during 12 h culture. **(A)** The K14-GFP transgene construct. **(B)** A schematic of the removal of the explants from both palatal shelves from each maxilla. **(C–H)** Fluorescence microscopic image showing oral views of the nasal septum at different developmental stages. At E15.0 **(C,F)**, after 12 h of culture **(D,G)**, after 24 h of culture **(E,H)**. GFP-positive epithelium was gathered at the anterior and median nasal septum (red arrowheads). **(F–H)** Higher magnification views in the region indicated by white boxes in **C–E**. Red arrowheads in **F–H** indicate the center of the developing nasal septum. White dotted lines in **C–E** indicate the posterior border of the primary palate. A, anterior; P, posterior; PP, primary palate; PS, secondary palate; NS, nasal septum. Scale bars: **C** 100 μm (**C–E** same magnification); **F** 100 μm (**F–H** same magnification).

In order to further investigate the behavior of epithelial cells of the developing nasal septum, transgenic mice that carry GFP under the promoter of *Keratin14* (*K14*) were used (Figure 2A) (Vaezi et al., 2002). Primary palate explants, including the nasal septum, were dissected at E15.0. The bilateral secondary palatal processes were further removed as the presumptive region of fusion could be directly visualized using a fluorescence microscope (Figure 2B). These dissected explants were captured at 12 and 24 h after culture. The uniform expression of GFP covering the nasal septum could be observed immediately after dissection (Figures 2C,F). After 12 h of culture, GFP-positive epithelium was gathered at the anterior and median nasal septum. Interestingly, accumulated GFP signaling could be detected in the region in which cellular protrusion was observed by SEM (Figures 2D,G). This accumulated GFP signaling remained after 24 h of culture (Figures 2E,H). These results indicate that the epithelial protrusion that was detected by SEM was caused by the behavior of the epithelial cells of the developing nasal septum.

### Live Imaging of Epithelial Cells of Nasal Septum Explants

**FIGURE 3:**
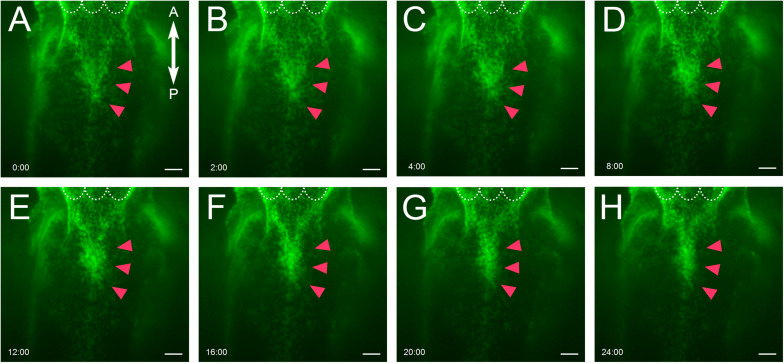
Live imaging of epithelial cells of nasal septum explants. **(A–H)** Epithelial cell movement captured by live imaging in the nasal septum explant model. At E15.0 **(A)**, after 2 h of culture **(B)**, 4 h of culture **(C)**, 8 h of culture **(D)**, 12 h of culture **(E)**, 16 h of culture **(F)**, 20 h of culture **(G)**, 24 h of culture **(H)**. The GFP-positive epithelium was slightly gathered at the anterior and median nasal septum (red arrowheads). White dotted lines indicate the posterior border of primary palate. A, anterior; P, posterior. Scale bars: 100 μm.

In order to assess the dynamism of the nasal septum epithelium during the fusion of nasal septum and the secondary palate, we used a live imaging technique. In this analysis, GFP-positive epithelium showed movement that gathered at the anterior and median nasal septum from 3 to 8 h of culture (Figures 3A–D). The converging cells showed a strong GFP signal. However, after that, dynamic cell migration did not occur until 24 h of culture and did not show the mesenchymal exposure that had been observed at the secondary palatal shelves (Figures 3E–H and Supplementary Movies 1, 2). Furthermore, the epithelial migration in the nasal septum was significantly shorter than that in the secondary palate (Supplementary Figure 1), indicating that the behavior of the epithelial cells of the nasal septum is distinguished from that of the secondary palate MEE under organ culture conditions, suggesting that different mechanisms underlie the removal of epithelial cells in each tissue.

### TUNEL Staining in Sections of Cultured Nasal Septum of K14-GFP Mice

**FIGURE 4:**
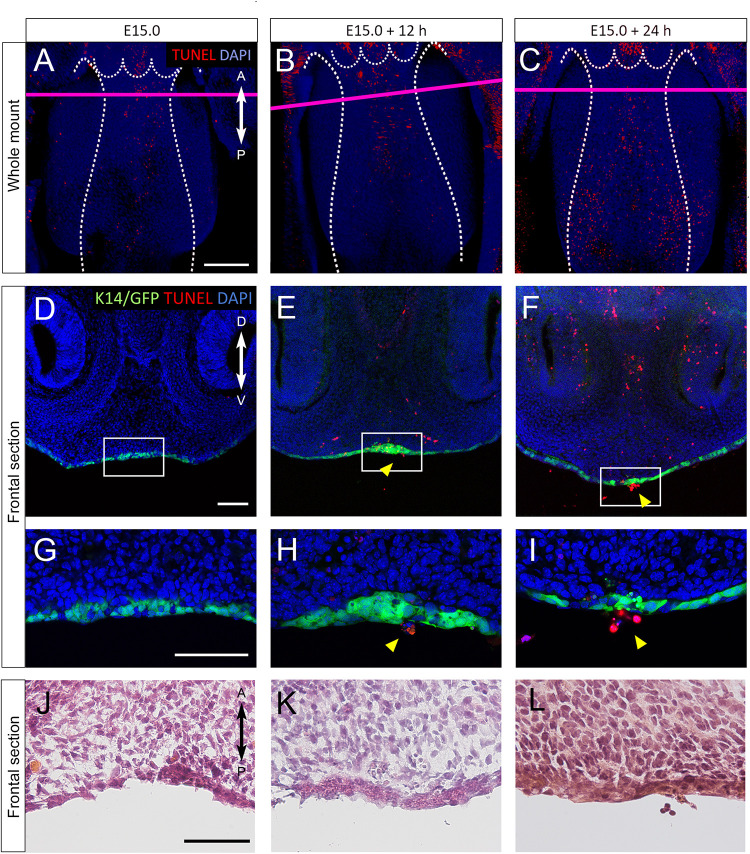
**(A–I)** TUNEL staining of sections of cultured nasal septum of *K14-GFP* mice. **(A–C)** A fluorescence microscopic image showing oral views of the nasal septum at E15.0 **(A)**, after 12 h of culture **(B)**, after 24 h of culture **(C)**. A, anterior; P, posterior. Scale bars: **A** 200 μm (**A–C** same magnification). **(D–I)** The frontal sections. **D–I** are from the area indicated by the red line in **A–C**. **(D–I)** Fluorescence microscopic images showing frontal views of the nasal septum at E15.0 **(D,G)**, after 12 h of culture **(E,H)**, after 24 h of culture **(F,I)**. **(G–I)** Higher magnification views in the region indicated by white boxes in **D–F**. Protrusion of epithelial cells of the nasal septum occurred (yellow arrowheads). **(J–L)** Hematoxylin and eosin staining images showing equivalent position of **G–I**. D, dorsal; V, ventral. Scale bars: **D** 100 μm (**D–F** same magnification); **G** 100 μm (**G–I** same magnification); **J** 50 μm (**J–L** same magnification).

In order to explore the cellular mechanism of nasal septum epithelium removal, we performed whole mount TUNEL staining to detect apoptotic cells during nasal septum development, since apoptosis is known to be one of the mechanisms by which the secondary palate MEE is removed. A small number of apoptotic cells could be seen at the nasal septum immediately after dissection at E15.0 (Figure 4A). An increased number of apoptotic cells at the anterior and median nasal septum were detected after 12 and 24 h of culture (Figures 4B,C). From the detailed observation of histological sections at the plane of the nasal septum and secondary palate fusion, we also detected apoptotic GFP-positive epithelial cells that showed extruding behavior (Figures 4D–I). The shape of the cells outside of the epithelium was round and differed from that of epidermal cells that have cell–cell adhesion. The region in which apoptosis was detected in this experiment corresponded to the region of the extruding epithelial cells that was observed in the SEM analysis. Hematoxylin and eosin staining of histological sections using cultured nasal septum showed a thickened epithelium (Figures 4J–L), which also corresponds to the region of strong GFP signaling in Figures 2, 3. We also confirmed that the *in vivo* epithelial cell death in the nasal septum increased during the fusion process between the nasal septum and secondary palate (Supplementary Figure 4). These results indicate that the cellular behaviors in our explant organ culture model were at least partially recapitulated the normal developmental process.

### Nasal Septum Phenotypes of *K14-Cre/Runx1*^fl/fl^ Mice

**FIGURE 5:**
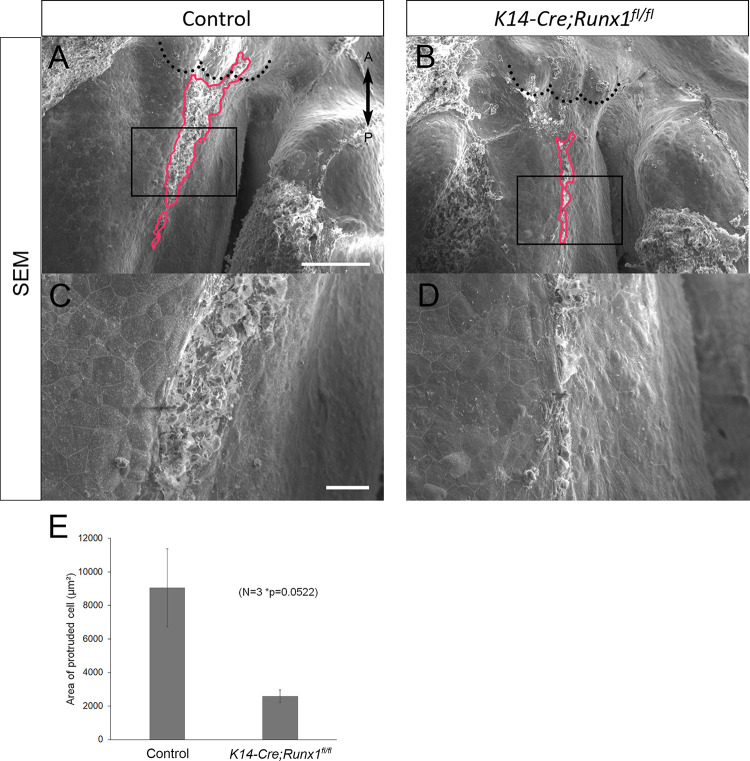
Nasal septum phenotypes of *K14-Cre/Runx1^fl/fl^* mice. **(A,B)** Occlusal views of the control and *K14-Cre/Runx1*^fl/fl^ mouse nasal septum at E15.0, after 12 h of culture. The area of epithelial protrusion in *K14-Cre/Runx1*^fl/fl^ mice was narrower than that in control mice (red line). **(C,D)** Higher magnification views of **(A,B)**. Black dotted lines in **A** and **B** indicate the posterior border of the primary palate. A, anterior; P, posterior. Scale bars: **A** 1000 μm (**A,B** same magnification); **C** 20 μm (**C**, **D** same magnification). **(E)** The area of epithelial protrusion was slightly reduced in *K14-Cre/Runx1*^fl/fl^ mice. *p = 0.0522, Student’s *t*-test, *n* = 3.

From our previous results, the epithelial-specific elimination of Runx1 is known to be exhibited at the anterior cleft palate (Sarper et al., 2018a,b). It is also known that fewer TUNEL-positive cells were detected on the unfused epithelium in *Runx1* mutants (Sarper et al., 2018b). We did not detect a significant reduction in the area of protruded cells in the cultured nasal septum of *K14-Cre/Runx1^fl/fl^* mice in comparison to the control group (Figure 5). While the statistical test did not support significance, there is a trend toward a reduced protruded area in *K14-Cre/Runx1^fl/fl^* mice that might become significant with a larger sample size. Furthermore, in *K14-Cre/Runx1^fl/fl^* maxillary complex cultures, the nasal septum epithelium showed a significant reduction in apoptosis in comparison with the control (Supplementary Figure 5).

Taken together, it is indicated that proper apoptotic behavior in the developing nasal septum is important for fusion with the secondary palate.

## Discussion

A time lapse observation of tissue explants *in vitro* provides solid evidence of cellular behavior (Bush and Jiang, 2012; Aoyama et al., 2019). Our time-lapse observation found that the epithelial extrusion of the nasal septum appeared at the presumptive region of palatal fusion and that this extruding region expanded in the rostral direction along the presumptive area of fusion. A TUNEL analysis demonstrated that these extruding cells were undergoing apoptosis. Since the secondary palatal process was removed, fusion does not occur at the primary and secondary palate junction in our culture system. The present findings indicated that extruding with apoptosis was initiated without contacting the secondary palatal shelf.

Our present findings demonstrated that cellular mechanisms in the initiation of palatal fusion differ between the primary/secondary palate junction and the bilateral secondary palate process junction. Apoptosis in palatal fusion is evident, both at the primary/secondary palate junction and at the bilateral secondary palate process junction; however, present results using live imaging of the GFP-labeled epithelium together with the maxillary explant culture system clarified the different initial cellular behavior between these junctions. Since the extrusion occurs at the specific region of the presumptive region of fusion, without contact with the opposing tissue, apoptosis would be initiated as an intrinsically programmed event at the primary/secondary palate junction. On the other hand, our previous *in vitro* study demonstrated that dynamic migration rather than apoptosis is initiated at the medial edge epithelium and that it is visible later on the secondary palate (Aoyama et al., 2019). The lack of influence of the opposing secondary palate is a limitation of this model; thus, it might not reflect the environment of fusion *in vivo*. However, our *in vitro* experimental models, with future pharmaceutical and genetic manipulations, could provide a potential tool to further investigate the initial cellular and molecular events of the palatal fusion and the difference in these events between the primary/secondary junction and the bilateral secondary palatal process. The limitations of this experimental method include the artifacts of explant culture. We confirmed continuous mesenchymal cell proliferation in maxillary complex cultures, which proves that this culture condition at least partially recapitulates the developmental process *in vivo* (Supplementary Figure 2). We also need to keep in mind that the fusion method for the nasal septum and secondary palate varies among species. For example, the mouse nasal septum only fuses with the secondary palate at the anterior-most point and leaves a common nasal passage, while the human nasal septum fuses along its entire length (Ferguson, 1978; Yu et al., 2017).

Our study demonstrated that apoptosis was initiated at the presumptive region of fusion as a programmed event at the primary/secondary palate junction. However, the functional significance of apoptosis remains controversial (Honarpour et al., 2001; Jin and Ding, 2006). The present and previous findings suggest that the induction of extrusion is critical for the fusion of facial processes and that it can be inhibited by *Runx1* depletion (Martínez-Alvarez et al., 2000; Takigawa and Shiota, 2004, 2007; Charoenchaikorn et al., 2009; Hu et al., 2015). Importantly, our previous report showed that the excision of *Runx1* in epithelial tissues takes place at the beginning of explant culture (Sarper et al., 2018a,b, 2019).

In Runx1 mutant mice, the anterior cleft palate is associated with reduced apoptosis and retained periderm, at the boundary between the primary and secondary palate junction. In these mutants, the expression of Tgfb3, a critical regulator of apoptosis, was significantly disturbed and TGFB3 protein rescued the formation of the anterior cleft (Sarper et al., 2018a,b, 2019). The molecular mechanism through which apoptosis is initiated remains unclear; however, the reduced induction of extrusion is associated with a reduction in the expression of *Tgfb3*. Of note, *Tgfb3* is widely expressed in the anterior palate; thus, the *Tgfb3* expression alone does not explain the region-specific induction of apoptosis and extrusion, suggesting that some other molecule is also involved in this regulatory process.

## Conclusion

The cellular and molecular mechanisms underlying nasal septum and secondary palate fusion are not completely understood. Our time-lapse observation using isolated primary palate explants combined with an SEM analysis revealed that the epithelial extrusion was initiated at the presumptive region of fusion. We also detected significant differences in the epithelial behavior between the fusing nasal septum and secondary palate. Our new experimental system could become a potential tool to further investigate the initial cellular and molecular events of palatal fusion and how these events differ between the primary/secondary palate junction and the bilateral secondary palatal process.

## Materials and Methods

### Animals

We utilized transgenic mice which express GFP under the control of the Cytokeratin-14 promoter (*K14-GFP*) (Vaezi et al., 2002) (Figure 2A). Mature female mice of C57BL/6J (CLEA, Tokyo, Japan) were mated with a *K14-GFP* male mouse. The day on which a vaginal plug was found was defined as embryonic day (E) 0.5. GFP expression was confirmed by the green fluorescent glow of the skin. For generating *K14-Cre/Runx1*^fl/fl^ mice, *K14-Cre/Runx1*^fl/+^ male mice were bred with *Runx1*^fl/fl^ female mice. Genotyping was performed using the genome DNA extracted from individual tail and conventional polymerase chain reaction (PCR), as previously described (Sarper et al., 2018b). The littermates that did not carry the *K14-Cre/Runx1*^fl/fl^ genotype were used as control animals. All of the animal experiments were performed in accordance with the guidelines of the Animal Care and Use Committee of the Osaka University Graduate School of Dentistry, Osaka, Japan. The protocol was recognized by the Committee on the Ethics of Animal Experiments of Osaka University Graduate School of Dentistry (permit number: 26-017-0, 26-004-0).

### Nasal Septum Explant Cultures

To evaluate the cellular behavior of nasal septum epithelium, we dissected the primary palate from E14.5 and E15.0 *K14-GFP* embryos for subsequent organ culturing and live imaging (Charoenchaikorn et al., 2009).

A rolling culture system (Ikemoto Scientific technology, Kanagawa, Japan) was utilized for organ culture, as previously described (Takigawa and Shiota, 2004). Dissected primary palate specimens were incubated in BGJb medium (Gibco@ Life Technologies) at 37°C in the glass bottle with a rotation speed of 25–30 rpm, in an atmosphere containing 50% O_2_, 5% CO_2_, and 45% N_2_ using a standard incubator for 12 or 24 h. The BGJb medium contains 0.2 mg/L D-Calcium pantothenate and 555.0 mg/L calcium lactate as calcium content.

For live imaging and the quantitative analysis, we removed both palatal shelves (Figure 2B). The nasal septum was cultured in a glass-bottomed dish (Matsunami, Osaka, Japan) with medium containing 0.6% low melting agarose (Wako Osaka, Japan). Explants were cultured and GFP was monitored using an all-in-one fluorescence microscope (BZ-X700, Keyence, Osaka, Japan).

### Imaging

(1) The morphological analysis by scanning electron microscope

The nasal septum was fixed in 2% glutaraldehyde and 2% paraformaldehyde in PBS with 100 nM Hepes for 6 h at 4°C. After washing with PBS, the explants were treated with 1% osmium tetraoxide for 1 h at room temperature (RT). Graded ethanol solution was used to dehydrate the samples. Explants were mounted on a specimen holder with carbon adhesion tape followed by platinum coating. All explants were examined by an SEM (JSM-6390LV, JEOL, Tokyo, Japan) with the secondary electron emission mode and accelerating voltages of 10 kV. The magnifications used were × 120, × 250, and × 800.

(2) Chronological analysis

All fluorescence images of explant cultures were captured by an all-in-one fluorescence microscope (BZ-X700, Keyence, Osaka, Japan) with filters for GFP channel (excitation: 475 nm, emission: 525 nm).

(3) Live imaging of primary palate cultures

Live imaging of explant cultures was performed using an all-in-one fluorescence microscope (BZ-X700, Keyence, Osaka, Japan), equipped with filters for GFP (excitation: 475 nm, emission: 525 nm) channel. The instrument was controlled by the BZ Viewer version 1.0 software program (Keyence, Osaka, Japan). Live images were captured with either ×10 0.45 NA objective lens or a ×20 0.75 NA objective lens to collect 26 Z-stacks (10.0 μm/step). The migration length of the epithelial cells was analyzed using 30 GFP-positive epithelial cells in the nasal septum and secondary palatal shelf, as previously described (Aoyama et al., 2019).

(4) TUNEL staining

Maxillary explants were fixed in 4% paraformaldehyde with 0.1 M sodium phosphate buffer (pH 7.4) overnight at 4°C. Whole-mount TUNEL staining was performed using fixed E15.0 palates and cultured palates. Histological sections of primary palate were produced as previously described. Frontal frozen sections (20 μm) were prepared from samples. The explants and sections were processed for a terminal deoxynucleotidyl transferase-mediated deoxyuridine triphosphate (dUTP) nick end labeling (TUNEL) assay using an In Situ Cell Death Detection Kit TMR Red (Roche Applied Science, IN, United States) according to manufacturer’s protocol. Finally, nuclear staining was performed using DAPI solution (Wako Pure Chemical Industries, Osaka, Japan).

## Data Availability Statement

All datasets generated for this study are included in the article/Supplementary Material.

## Ethics Statement

The animal study was reviewed and approved by the Committee on the Ethics of Animal Experiments of Osaka University Graduate School of Dentistry.

## Author Contributions

SY performed the experiments and wrote the manuscript. HK funded the study, performed the experiments, and wrote the manuscript. JM, GA, SS, AO, TI, KN, and YU performed the experiments. ST provided technical support and conceptual advice. TY supervised the experimental analysis, edited the manuscript, and supplied funds. All authors discussed the results and implications and commented on the manuscript at all stages.

## Conflict of Interest

The authors declare that the research was conducted in the absence of any commercial or financial relationships that could be construed as a potential conflict of interest.

## Supplementary Material

Supplementary Movie 1 Live imaging of the epithelial cells of nasal septum explants. Scale bar: 100 μm. Images were captured every 15 min for 24 h.

Supplementary Movie 2 Live imaging of the epithelial cells of secondary palate explants. Scale bar: 100 μm. Images were captured every 10 min for 8 h.

Supplementary Figure 1 The migration length was significantly increased in the secondary palate. ^∗^*p* < 0.05, Student’s *t*-test, *n* = 30.

Supplementary Figure 2 The E-cadherin and PHH3 expression in the nasal septum. D, dorsal; V, ventral. Scale bars: 100 μm.

Supplementary Figure 3 The size of the cultured tissue at E 15.0. The approximate width is 1500 μm. The approximate length is 1000 μm. The approximate thickness is 700 μm. A, anterior; P, posterior. Scale bars: **A** 500 μm (**A**, **B** same magnification).

Supplementary Figure 4 TUNEL staining of the frontal section of the nasal septum at E14.5 and E15.5. D, dorsal; V, ventral. Scale bars: **A** 50 μm (**A**, **B** same magnification). **(C)** The numbers of TUNEL-positive cells were significantly increased at E15.5. ^∗^*p* < 0.05, Student’s *t*-test, *n* = 3.

Supplementary Figure 5 TUNEL staining of the frontal section of the control and *K14-Cre/Runx1*^fl/fl^ mouse nasal septum. D, dorsal; V, ventral. Scale bars: **A** 50 μm (**A**, **B** same magnification). **(C)** The numbers of TUNEL-positive cells were significantly decreased at *K14-Cre/Runx1*^fl/fl^ mouse. ^∗^*p* < 0.05, Student’s *t*-test, *n* = 3.
