# Supplementary figures and images for: Observation of the Epithelial Cell Behavior in the Nasal Septum During Primary Palate Closure in Mice
Source: Front Physiol. 2020 Oct 2;11:538835. doi: 10.3389/fphys.2020.538835 (PMC7566916; doi:10.3389/fphys.2020.538835)

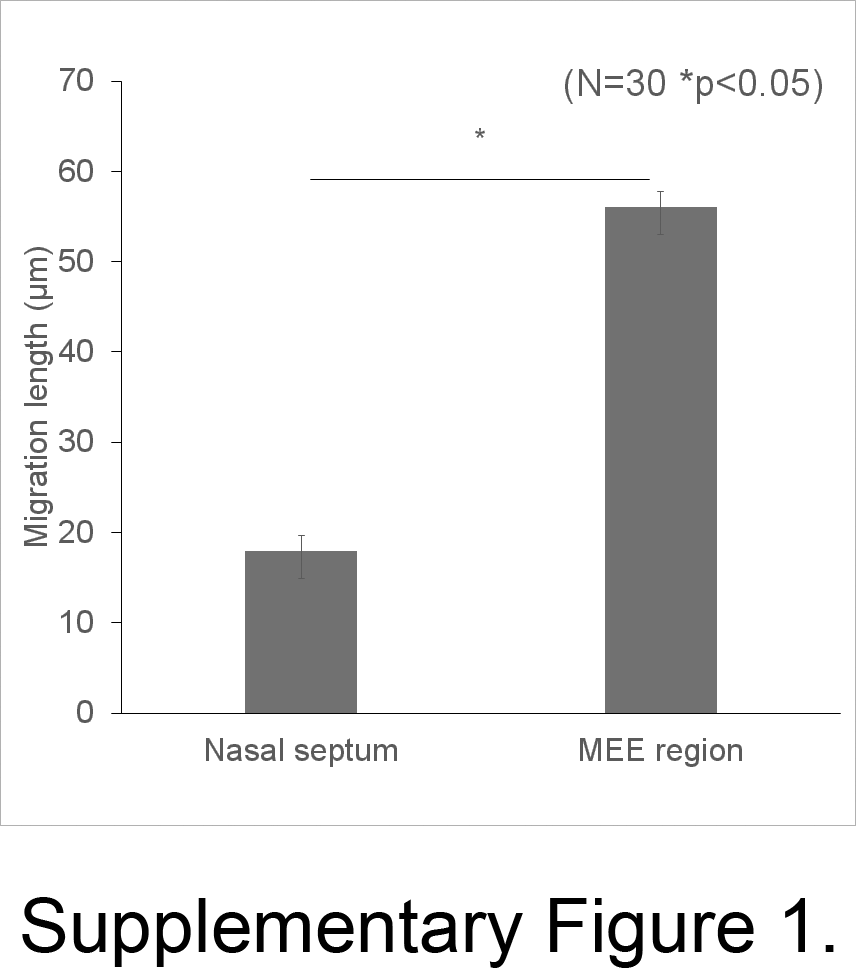

Supplement: Supplementary Figure 1 — The migration length was significantly increased in the secondary palate. ∗p < 0.05, Student’s t-test, n = 30. [file Image_1.TIF]

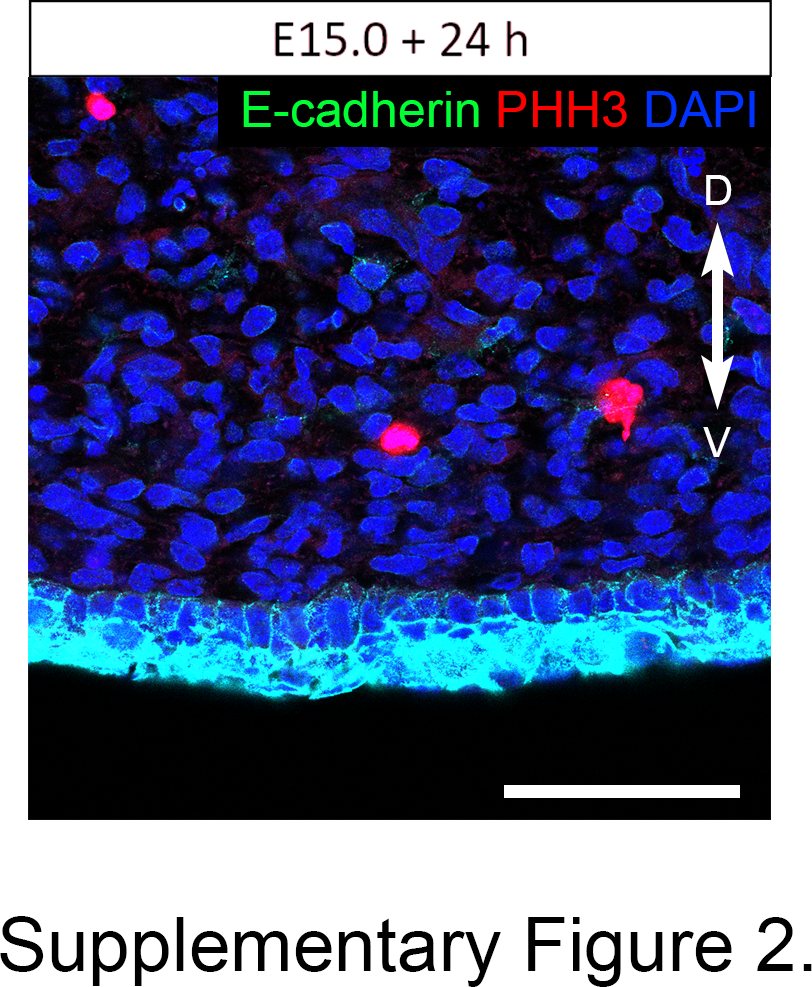

Supplement: Supplementary Figure 2 — The E-cadherin and PHH3 expression in the nasal septum. D, dorsal; V, ventral. Scale bars: 100 μm. [file Image_2.TIF]

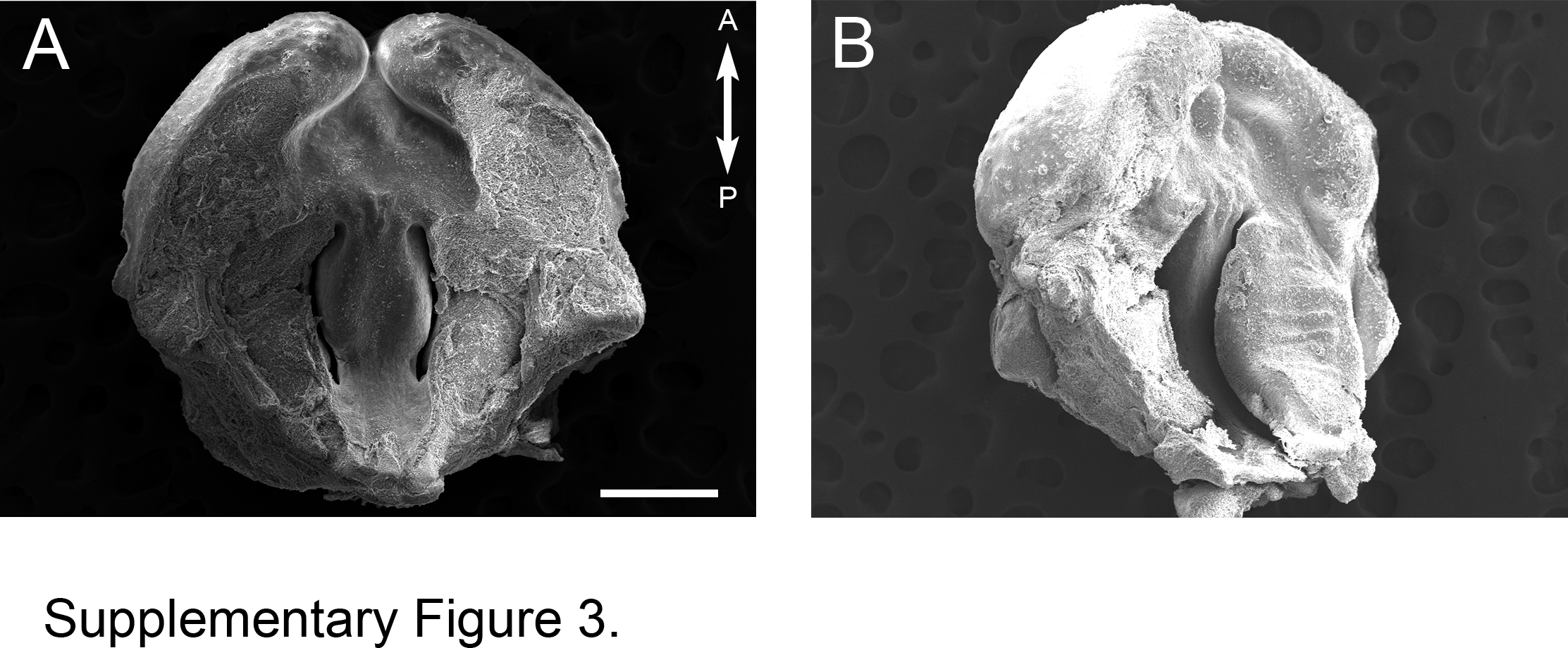

Supplement: Supplementary Figure 3 — The size of the cultured tissue at E 15.0. The approximate width is 1500 μm. The approximate length is 1000 μm. The approximate thickness is 700 μm. A, anterior; P, posterior. Scale bars: A 500 μm (A, B same magnification). [file Image_3.TIF]

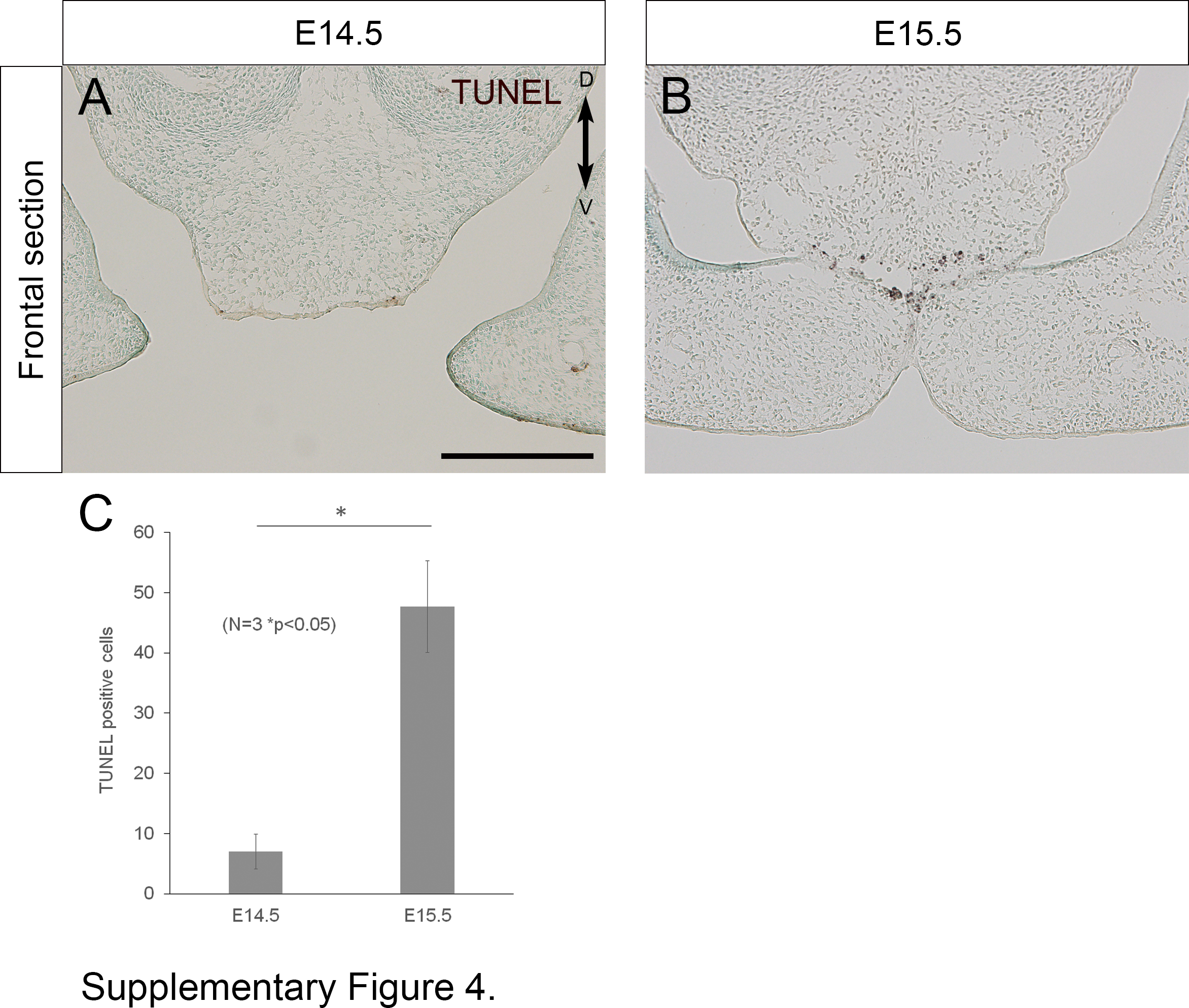

Supplement: Supplementary Figure 4 — TUNEL staining of the frontal section of the nasal septum at E14.5 and E15.5. D, dorsal; V, ventral. Scale bars: A 50 μm (A, B same magnification). (C) The numbers of TUNEL-positive cells were significantly increased at E15.5. ∗p < 0.05, Student’s t-test, n = 3. [file Image_4.TIF]

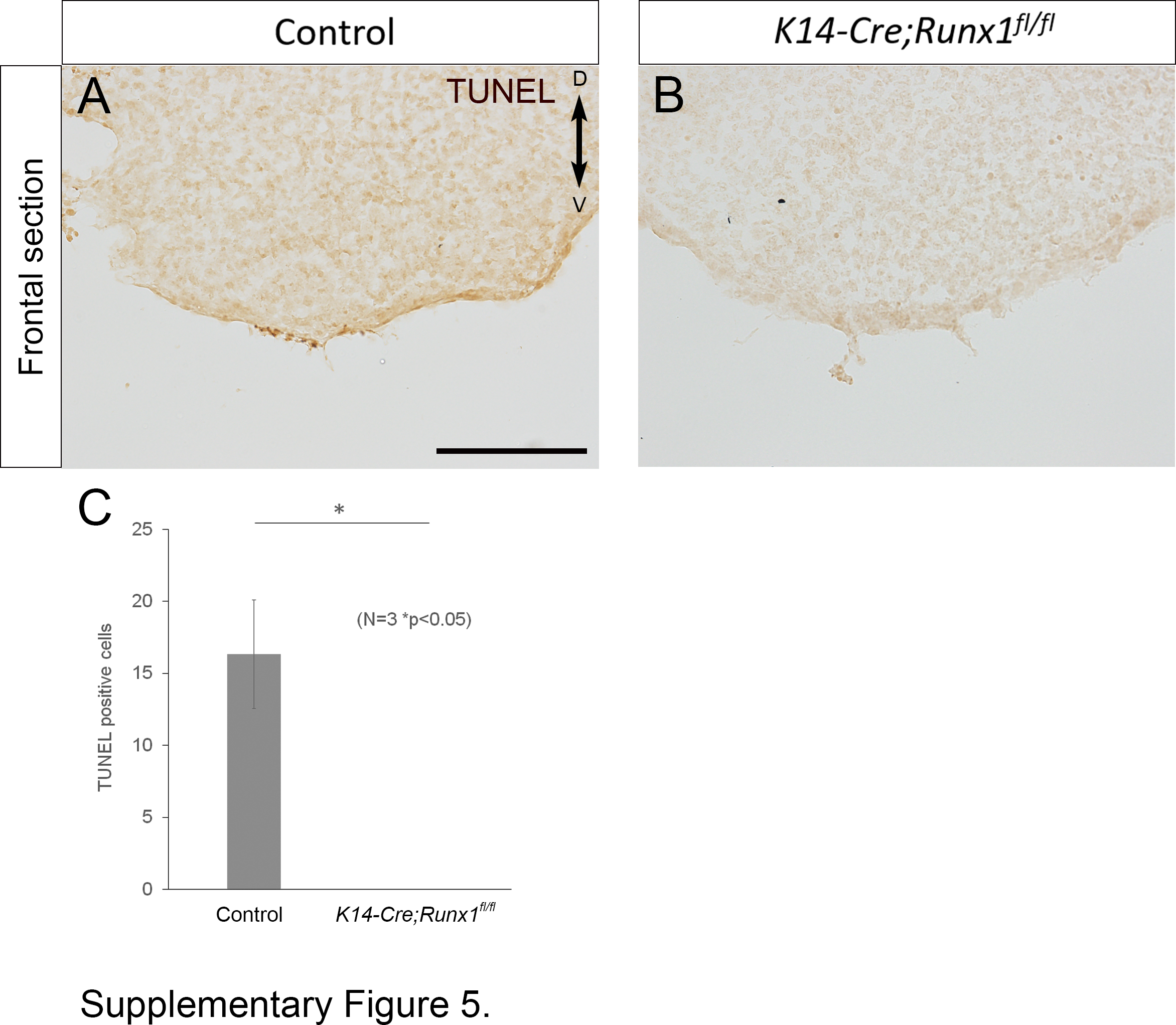

Supplement: Supplementary Figure 5 — TUNEL staining of the frontal section of the control and K14-Cre/Runx1fl/fl mouse nasal septum. D, dorsal; V, ventral. Scale bars: A 50 μm (A, B same magnification). (C) The numbers of TUNEL-positive cells were significantly decreased at K14-Cre/Runx1fl/fl mouse. ∗p < 0.05, Student’s t-test, n = 3. [file Image_5.TIF]
